# Parallel reorganization of protein function in the spindle checkpoint pathway through evolutionary paths in the fitness landscape that appear neutral in laboratory experiments

**DOI:** 10.1371/journal.pgen.1006735

**Published:** 2017-04-14

**Authors:** Alex N. Nguyen Ba, Bob Strome, Selma Osman, Elizabeth-Ann Legere, Taraneh Zarin, Alan M. Moses

**Affiliations:** 1 Department of Cell & Systems Biology, University of Toronto, Toronto, Ontario, Canada; 2 Center for Analysis of Evolution and Function, University of Toronto, Toronto, Ontario, Canada; 3 Department of Ecology and Evolutionary Biology, University of Toronto, Toronto, Ontario, Canada; Washington University School of Medicine, UNITED STATES

## Abstract

Regulatory networks often increase in complexity during evolution through gene duplication and divergence of component proteins. Two models that explain this increase in complexity are: 1) adaptive changes after gene duplication, such as resolution of adaptive conflicts, and 2) non-adaptive processes such as duplication, degeneration and complementation. Both of these models predict complementary changes in the retained duplicates, but they can be distinguished by direct fitness measurements in organisms with short generation times. Previously, it has been observed that repeated duplication of an essential protein in the spindle checkpoint pathway has occurred multiple times over the eukaryotic tree of life, leading to convergent protein domain organization in its duplicates. Here, we replace the paralog pair in *S*. *cerevisiae* with a single-copy protein from a species that did not undergo gene duplication. Surprisingly, using quantitative fitness measurements in laboratory conditions stressful for the spindle-checkpoint pathway, we find no evidence that reorganization of protein function after gene duplication is beneficial. We then reconstruct several evolutionary intermediates from the inferred ancestral network to the extant one, and find that, at the resolution of our assay, there exist stepwise mutational paths from the single protein to the divergent pair of extant proteins with no apparent fitness defects. Parallel evolution has been taken as strong evidence for natural selection, but our results suggest that even in these cases, reorganization of protein function after gene duplication may be explained by neutral processes.

## Author summary

Parallel evolution of protein domain organization following gene duplication has been demonstrated in the spindle checkpoint pathway leading to the hypothesis that this organization is likely to be adaptive. We test this hypothesis by reconstructing budding yeast strains with a spindle checkpoint pathway containing a protein with ancestral domain organization, and systematically perform stepwise duplication, degeneration and complementation of the duplicated protein. We show that, under laboratory conditions where the spindle checkpoint pathway is necessary for growth, degeneration of the ancestral pathway organization to the extant sub-functionalized proteins is consistent with a neutral model of duplication-degeneration-complementation.

## Introduction

Gene duplication and sub-/neo-functionalization are processes that both increase genomic complexity and are thought to be the major sources of genetic novelty in organisms [1]. Models seeking to explain the retention of paralog genes include relaxation of selection constraints leading to functional novelty through transcriptional changes, splicing divergence or functional repartitioning of protein domains. Particularly interesting are cases of repeated, parallel evolution of these paralog genes that has been taken to be strong evidence for natural selection (several examples reviewed in [2]). In most model organisms, the spindle checkpoint pathway includes the paralogous Bub1 and Mad3 proteins. Surprisingly, although Bub1 and Mad3 are highly diverged, it was recently reported that this paralog pair has arisen from at least nine independent duplication events throughout the tree of life [3]. Perhaps even more striking is that in each event, the duplication almost always leads to a Bub1 homolog, containing a kinase domain and a Mad3 homolog containing a pseudo-substrate APC inhibitor motif [3]. This reorganization of protein function has therefore been thought to be adaptive, leading to a more complex spindle checkpoint pathway [3,4]. However, theoretical work suggests that a causal link between increased genetic complexity and adaptation may not be as clear as commonly assumed [5,6]. For example, if degeneration and complementation of the ancestral bi-functional protein must always lead to Bub1 and Mad3 division of function (i.e., kinase function separated from the pseudo-substrate inhibitor motif), then the repeated observation of Bub1 and Mad3 protein organization might be due to a rate of degeneration that is higher than the rate of reconstitution.

Whether sub-functionalization of a bi-functional protein is adaptive or neutral cannot be easily distinguished by sequence analysis alone. Both models predict complementary conservation of function. However, precise quantitative fitness measurements in organisms with short generation time, such as yeasts, can be used to address questions about these two models of sequence evolution (adaptive vs neutral). Using comparative approaches that delineate functional regions of proteins, we found that this gene duplication leads to sequence signatures that indicate repartitioning of the ancestral function in the extant paralogs. To test whether this increase in complexity (from a pathway containing a bi-functional gene to a pathway containing two specialized genes) in the spindle checkpoint is adaptive, we employ high-throughput quantitative fitness measurements in laboratory conditions stressful for the spindle checkpoint pathway and we systematically dissect the reorganization in protein architecture of an ‘ancestral’ bi-functional gene. To our surprise, we could not detect any increase in fitness for the stepwise functional reorganization (duplication, degeneration and complementation (DDC)).

## Results

### Subfunctionalization in the spindle checkpoint network

Preservation of duplicate genes as explained by the duplication-degeneration-complementation model is a neutral process by which repartitioning of the functions of the ancestral protein occurs within the two extant duplicates [7]. Although sub-functionalization is a neutral process, it can lead to adaptation in the case of adaptive conflicts: mutations that are precluded from occurring in the ‘ancestral’ gene but are adaptive when the functions of the ancestral gene are separated. This resolution of adaptive conflicts can sometimes explain the fitness benefit of mutations that ‘specialize’ multifunctional proteins [8]. Nevertheless, complementation by functional repartitioning or complete functional preservation in one of the two copies is still expected in the cases where mutations leading to neo-functionalization have occurred.

**Fig 1:**
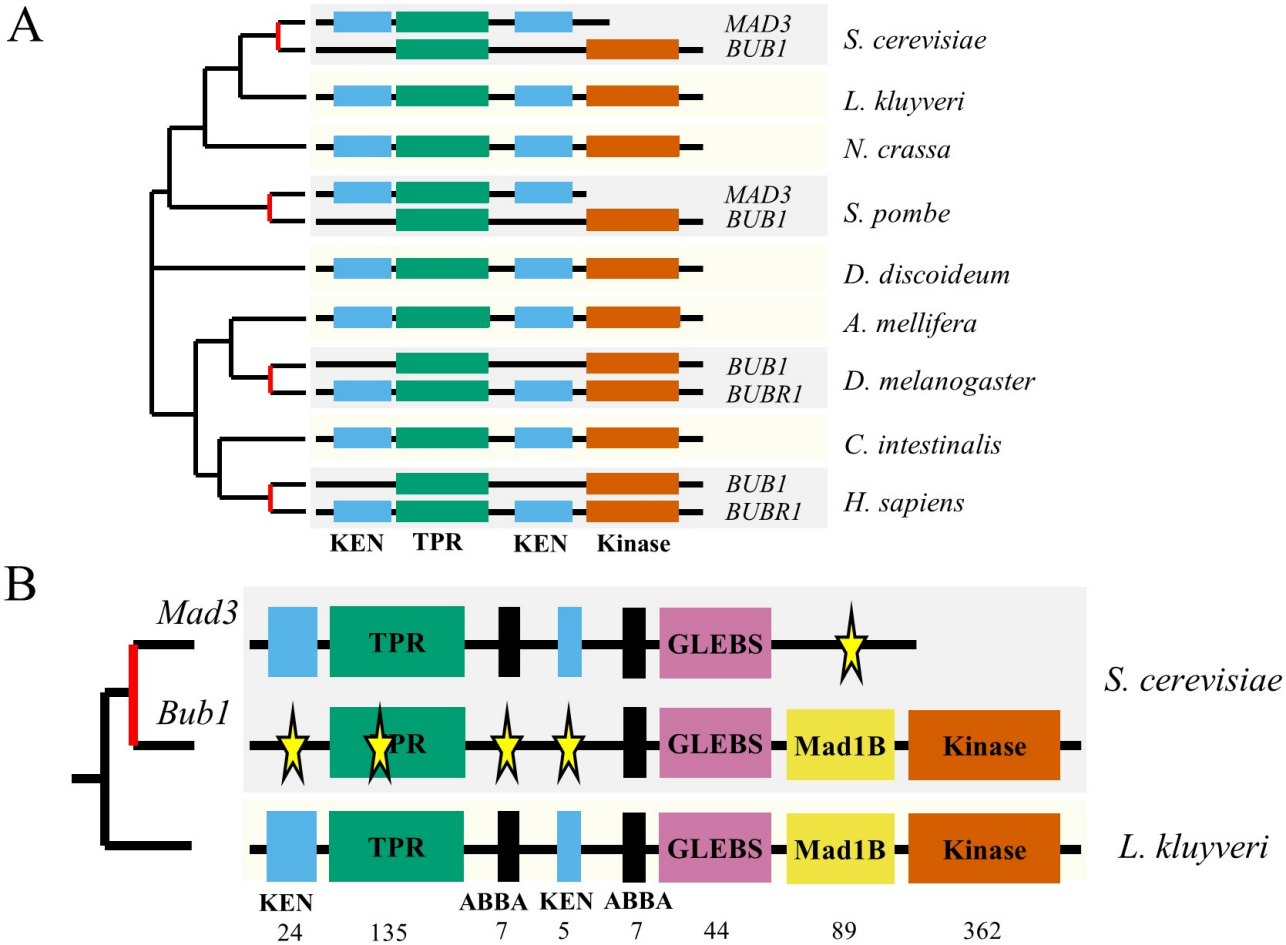
Sequence analysis of the Mad3/Bub1 paralogs. A) Independent duplication events in the bi-functional gene lead to similar domain arrangements. Red line on the phylogenetic tree indicates an inferred duplication event (species highlighted in dark grey). Several species retain the bi-functional gene (species highlighted in light grey). We note that duplicate protein with the KEN boxes in human has been shown to contain a pseudokinase and that this ‘MAD3’-like homolog has been named BUBR1 in higher eukaryotes. Schematics are not to scale. B) Amino-acid resolution analysis reveals several regions with changes in constraints in both Bub1 and Mad3 proteins. KEN: KEN-box, TPR: Tetratricopeptide-domain, ABBA: Cdc20 binding motif, GLEBS: Gle2-binding-sequence-domain (binds Bub3), MAD1B: Mad1-binding region, Kinase: Kinase-domain. Numbers indicate the amino acid size of each segment of *L*. *kluyveri*. The whole protein is 982 amino acids. Stars indicate detected changes in evolutionary constraints.

It has previously been shown that Bub1 and Mad3 protein reorganization had occurred several times over the eukaryotic tree of life, leading to similar sequence profiles (Fig 1A, and see [3]). To obtain an amino acid resolution view of the evolution of the paralogous proteins, we performed sequence analysis of the duplication that occurred in the whole-genome duplication of budding yeasts ([9], see Methods). Briefly, a phylogenetic hidden Markov model is used to identify short or large regions of conservation relative to the flanking amino acid sequence. This algorithm is applied to single copy proteins from species that diverged before the duplication to identify short motifs or domains that are under selective constraints. We then map these regions to the duplicates and test whether there is evidence of relaxation in constraints in the clade post-duplication using likelihood ratio tests. This analysis revealed several protein regions, in addition to the KEN boxes and kinase domain identified in previous studies [3], that have repartitioned from the ancestral sequence to the paralogs (Fig 1B).

This more detailed view of the changes in constraints allowed us to propose why some of the changes were correlated (S1A Fig for an example). For example, Mad3 is known to interact with Cdc20 via its N-terminal KEN box motif and its tetracopeptide repeat domain (TPR) [10]. According to 3D structural information of Mad3 (S1B Fig), specific residues in the TPR domain (shown in yellow in S1B Fig) appear to contact the KEN box (shown in red in S1B Fig) and may stabilize the interaction of Mad3 with Cdc20 (S1C Fig) [11]. Bub1, on the other hand, does not possess the N-terminal KEN box but still requires the TPR for proper function [12]. We can detect changes in constraints on these specific residues of the TPR of Bub1, suggesting that the degeneration of residues in the TPR domain or the KEN box will disrupt the same function (binding to Cdc20) and that loss of selection constraints on that function will lead to degeneration of both. Indeed, the same correlated changes in constraints exemplified here are also observed in the mammalian, drosophila, and fission yeast Bub1 and Mad3, which occurred through independent gene duplication events (S1D Fig). The changes in constraints at these specific residues are unlikely to disrupt the other functions of the TPR.

On the other hand, the pattern of evolution on the ABBA motifs in the yeast paralogs is more complicated. The ABBA motif is known to be required for binding to Cdc20 [13], and indeed, both Bub1 and Mad3 retain at least one copy of the ABBA motif (Fig 1B). Nevertheless, we can clearly detect motif turnover in the first ABBA motif in Bub1 (Fig 1B). This suggests two possible explanations: 1) the two ABBA motifs serve the same function and the loss of the first motif was compensated in the Bub1 protein by another motif, 2) the two ABBA motifs may bind Cdc20 for different functional reasons and the Bub1 protein underwent a single change in constraint on this motif. Since we have not identified a newly conserved motif in the Bub1 protein (which could compensate for the loss of the first ABBA motif), we believe that the second possibility is more parsimonious.

Consistent with important functions for the domains identified in the ancestral fungal protein, there were no conserved regions that were lost in both paralogs (Fig 1B). The repartitioning of functional regions suggests that the duplication lead to sub-functionalization. We therefore sought to experimentally verify that sub-functionalization had occurred in the paralog pair. As a proxy for the ‘ancestral gene’, we obtained the gene from *Lachancea kluyveri* which diverged prior to the whole-genome duplication event (see Discussion). We refer to this gene as the ‘single-copy protein (SCP)’, and transformed it into *S*. *cerevisiae*.

**Fig 2:**
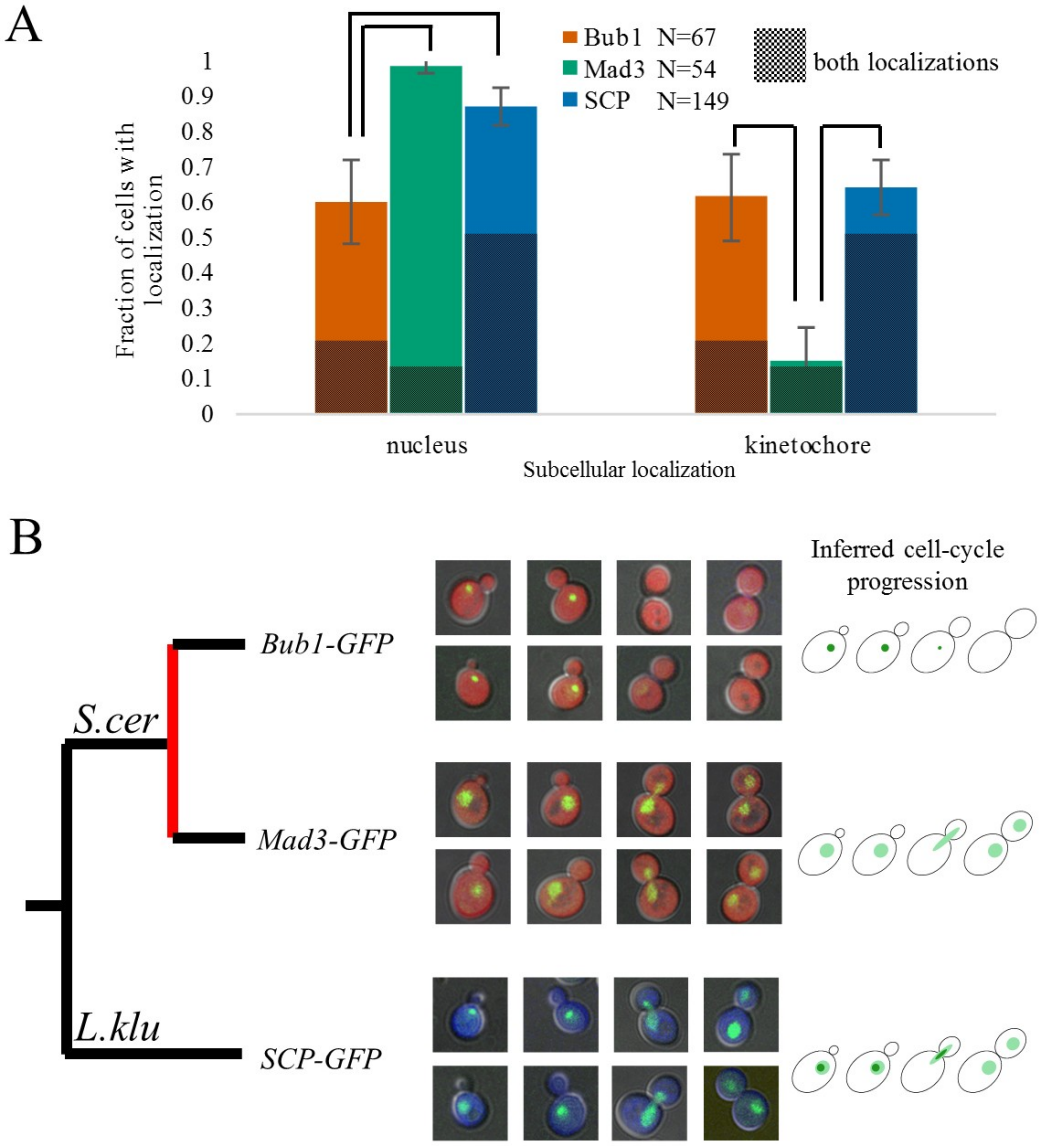
Localization of the Bub1/Mad3 paralogs. A) Green-fluorescent protein tagged gene cloned from *L*. *kluyveri* (single-copy protein, SCP) localizes differently than either the Bub1 or Mad3 proteins from *S*. *cerevisiae*. In both cases, the single-copy protein is in a background expressing a cytoplasmic blue fluorescent protein, while the *S*. *cerevisiae* protein is in a background expressing a red fluorescent protein. Quantification of the localization pattern was performed by visual inspection of the green fluorescent protein of cells grown in co-culture, and then the strains were classified according to their blue or red fluorescence. Cells without obvious GFP localization were discarded prior to the analysis (in total 26 cells were discarded from the Bub1-GFP strain, 7 from the Mad3-GFP strain, and 11 from the SCP-GFP strain). The shaded section of the bars represents cells for which we found localization in both nucleus and kinetochore puncta and are therefore present in both bar graphs. Error bars represent 95% confidence intervals of the fraction as obtained by bootstrap replicates. Lines over the bar chart represent p<0.05 (Fisher’s exact test corrected for multiple testing). B) Green-fluorescent protein tagged genes cloned from *L*. *kluyveri* (SCP) localizes as a mixture of Bub1 and Mad3 protein from *S*. *cerevisiae*. Bub1 is clearly visible as puncta in S/G2 phase of the cell cycle, presumably when it localizes to the kinetochores, while Mad3 is seen as nuclear throughout the cell-cycle. The single-copy protein is also seen as nuclear throughout the cell-cycle, however is also seen as puncta in S/G2.

We first assessed the localization of the single-copy protein because it was known that Bub1 and Mad3 localize to different subcellular compartments: Bub1 is localized to the kinetochores in a Mps1 dependent manner during specific phases of the cell-cycle [14] and Mad3 is constitutively localized in the nucleus [15]. If Bub1 and Mad3 were products of a sub-functionalization event, we would also expect the *L*. *kluyveri* protein to localize to both subcellular compartments. To test this, we tagged the three proteins with GFP and visualized their localization by fluorescence microscopy. To account for possible differences in growth or imaging conditions that may influence the apparent localization of the proteins, we designed an assay where the tagged single-copy protein could be visualized alongside the *S*. *cerevisiae* tagged protein in the same field of view using the identical GFP tag. Briefly, we expressed additional cytoplasmic fluorescent proteins of other colors (non-GFP) that allow us to differentiate which cells carried the *L*. *kluyveri* protein fusion or the *S*. *cerevisiae* fusion and obtained fluorescent micrographs of mixed cultures (see Methods). As expected, we observed a distinct localization pattern for the single-copy protein, which was quantitatively different from either Bub1 or Mad3 (Fig 2A). Upon closer inspection, we found that the localization of the single-copy protein appears to be a mixture of the localization patterns of Bub1 and Mad3. Consistent with this, when ordering by bud size as marker of cell stage, we observed that the *L*. *kluyveri* protein localized constitutively to the nucleus, and also showed a punctate localization within the nucleus in the early cell-cycle (Fig 2B).

**Fig 3:**
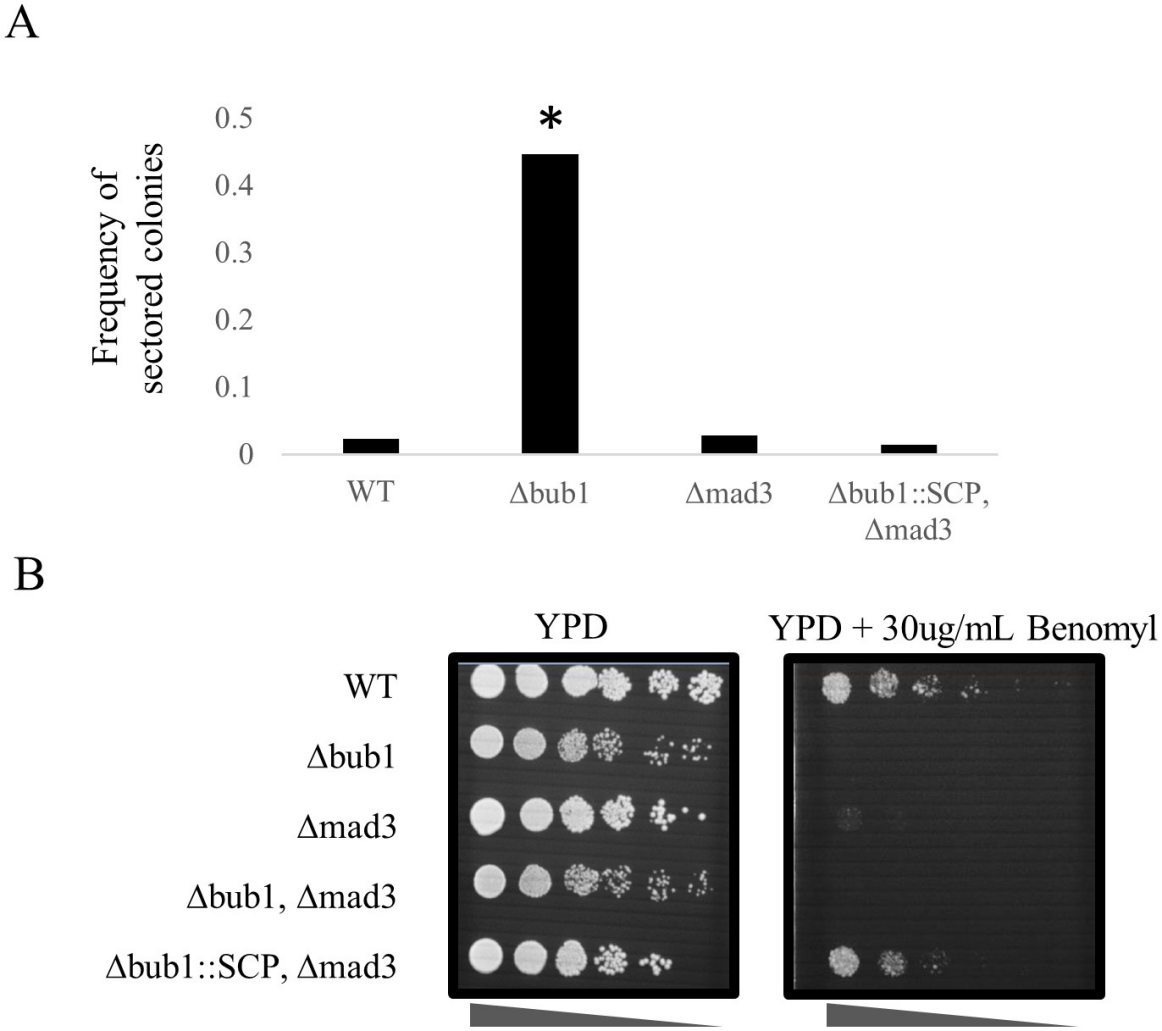
Phenotype rescue of spindle checkpoint mutants. A) Yeast cells carrying the non-essential chromosome loss reporter were plated on agar plates with low adenine supplementation. The relative increase in sectored colonies after two days of growth (sectored colonies/white colonies) is indicative of higher chromosome loss rate, which is a phenotype easily visible in cells lacking Bub1. In contrast, cells carrying the SCP gene have a loss rate that is not distinguishable from wild-type. * indicates statistical significance P<0.001. B) Yeast cells expressing the single-copy protein (SCP) at the BUB1 locus can grow on YPD plates with 30ug/mL benomyl even in the absence of Mad3, as assessed by a 10-fold spot dilution assay. YPD plate was imaged after 2 days. YPD plate containing benomyl was imaged after 3 days.

Because Bub1 and Mad3 are required for the spindle-checkpoint pathway, we tested whether the single-copy protein could rescue defects of cells lacking Bub1 or Mad3 with respect to the functionality of the pathway. To do so, we took advantage of a strain carrying a non-essential chromosome containing the ochre suppressor SUP11 [16]. This strain forms red-pigmented colonies upon loss of this chromosome due to the presence of the ade2-101 allele. As has been reported previously [17], we found that strains without a functional Bub1 gene have a very strong increase in chromosome loss rate (29/65 vs 3/131 sectored colonies, p-value < 0.0001). However, we were unable to detect an increase in chromosome loss rate for strains lacking a functional Mad3 gene (5/177 vs 3/131 sectored colonies). Nevertheless, cells lacking Bub1 and Mad3, but carrying the single-copy gene driven by the Bub1 promoter in this strain completely abolished the Bub1 chromosome segregation fidelity defect (3/207 vs 3/131 sectored colonies, Fig 3A). We next sought to verify if the single-copy protein could rescue the growth defects of impaired cell-cycle functions of cells lacking Bub1 and Mad3. Cells lacking Bub1 or Mad3 are highly sensitive to benomyl, a microtubule destabilizing drug [18], because cells fail to correctly detect mitotic spindle attachments. If the single-copy protein can perform the functions of both Bub1 and Mad3, we expect that the single-copy protein would rescue the fitness defect of cells lacking Bub1 or Mad3. If the phenotype is not fully rescued, it may indicate neo-functionalization and adaptation in the Bub1 or Mad3 protein (or it may be due to an artifact of expressing a heterologous gene). To test this, we performed spot dilution assays and found that, if expressing the single-copy protein, cells lacking Bub1 and Mad3 can grow in the presence of benomyl with similar growth characteristics to wild-type *S*. *cerevisiae* cells (Fig 3B). We could not simply test the function of the spindle checkpoint in other species as we observed that yeasts other than *S*. *cerevisiae* were highly resistant to benomyl (S2 Fig).

At the limits of the resolution of this plate assay (we estimate that this assay can detect at the minimum only a 5% growth difference), and taken together with the localization data, these results suggest that the single copy protein can rescue Bub1 and Mad3 function in *S*. *cerevisiae*. Consistent with the DDC model, the sub-functionalization of the Bub1 and Mad3 proteins may confer no fitness advantage to *S*. *cerevisiae*.

### A precise and rapid quantitative fitness assay for gene network rewiring

**Fig 4:**
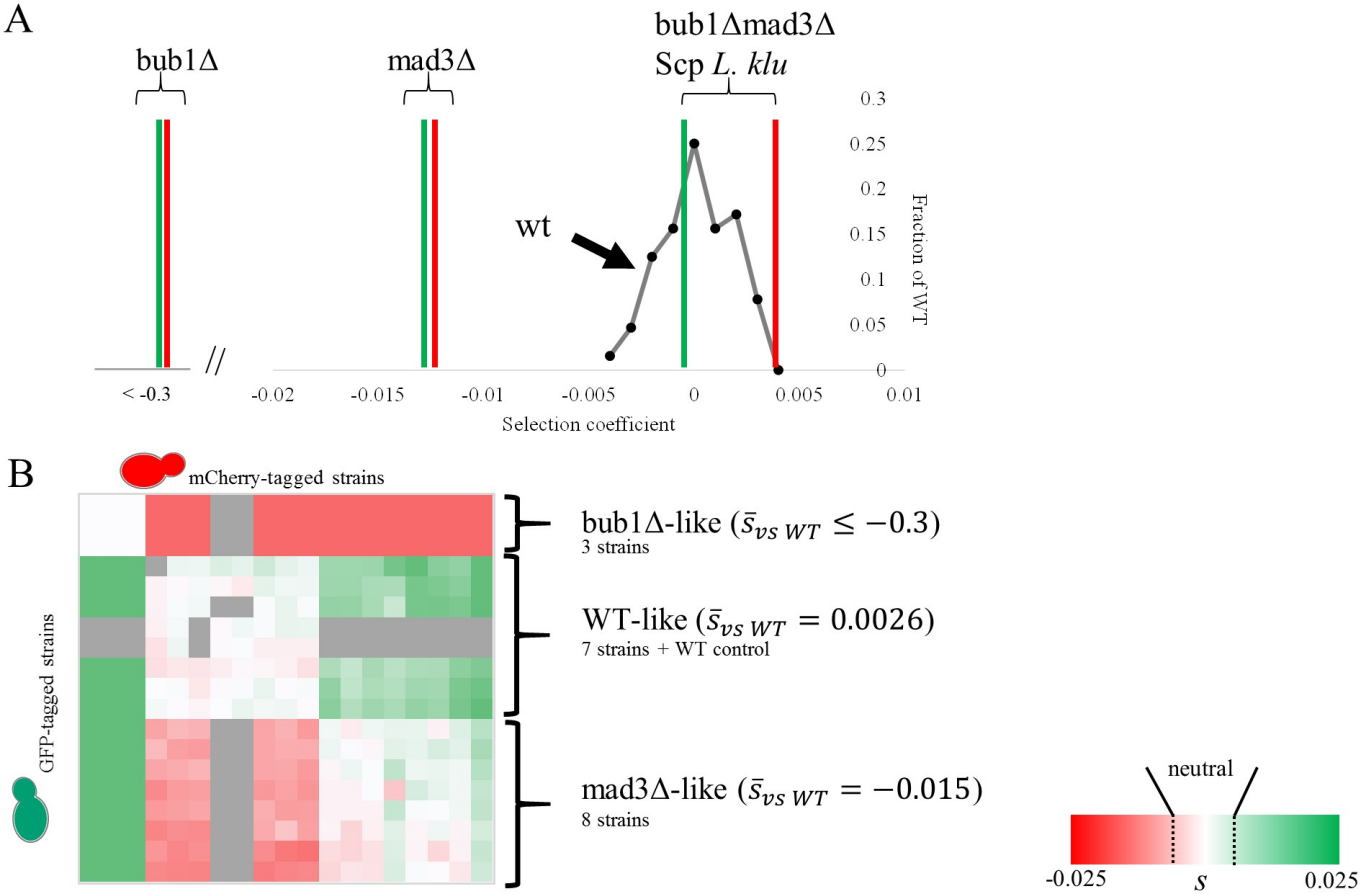
Competitive fitness measurements of reconstructed genotypes. A) The resolution of our assay was assessed by performing 64 competitions of wild-type spores from the SGA process carrying identical marked alleles from a mosaic of BY4741/BY4742 parental background and are assayed using our high-throughput fitness assay (grey curve). We indicate the measured fitness effects of strains carrying mad3Δ, bub1Δ, or the single-copy protein at the *BUB1* locus as red and green vertical bars. The two bars for each genotype represent measurements of relative selection coefficient to the wild-type control from replicate strains carrying swapped fluorescent proteins. B) Heat map clustering of all the competitive fitness assays using Euclidean distance and average linkage of the selection coefficients. The transpose off-diagonals are biological replicates with strains carrying swapped fluorescent proteins. Three major fitness classes are observed, which correspond to genotypes that have fitness comparable to mad3Δ cells, bub1Δ cells, or to the wild-type (as indicated by the mean selection coefficient measured for the strains present in each cluster). Grey cells in the heat map indicate competitions that were not performed.

It is possible that the spot dilution assay did not have enough resolution to detect the fitness advantage of the reorganization of protein function in the paralogs. To address this, we designed a method to more precisely quantify relative selection coefficients (*s*, see Methods). Briefly, our assay is a competitive fitness assay that takes advantage of flow-cytometry to provide relative counts of fluorescently labeled cells within a growing population [19] and this assay can be performed in a high-throughput fashion where the combination of alleles of interest are tested in cellular backgrounds expressing different fluorescent proteins as experimental replication. Replicate strains can be rapidly created by the SGA cloning strategy (see Methods and [20], S3 Fig). To determine the resolution limit of this fitness assay, we generated 16 spores from a cross between a strain carrying a wild-type Bub1 allele marked with CaURA3 (the *URA3* gene from *Candida albicans*, which complements the *URA3* gene from *S*. *cerevisiae*) and a query strain (containing SGA mating type reporters) expressing a green or a red fluorescent protein (8 spores for each cross). These spores were competed to form 64 fitness assays. We reasoned that if any additional single nucleotide polymorphisms (SNPs) between the query strains and parental strain of our mutant arrays had a detectable fitness effect (but masked due to epistasis within their respective backgrounds), or if these SNPs showed non-transitive fitness effects, they would be uncovered within these 16 spores. We compared the relative proportions of cells expressing the green fluorescent protein and red fluorescent protein at the 20^th^ generation to the 40^th^ generation and we calculated the selection coefficient for each competition (see Methods). Because our strains are supposedly genetically identical (except for the possibility of non-shared SNPs), we expect an average selection coefficient of zero and the standard deviation obtained from this test can be used to estimate the resolution of our assay (deviations due to growth conditions or to the non-shared SNPs). We measured the selection coefficients of the wild-type strains and observed a mean selection coefficient of 0.00069, with a standard deviation of 0.0017 (Fig 4A). This indicates that the resolution of our assay is in the order of *s* = 0.0033 (1.96 times the standard deviation) and we believe this represents the difference in growth rate that we can detect. To account for other possible variations that may occur during the course of the study (changes in media, etc) we therefore chose to report as deleterious/beneficial any differences in fitness where both replicates of a competition exceeded a selection coefficient with an absolute value of 0.005 or greater while remaining consistent with all other competitions.

We next compared strains carrying deletions of Bub1 or Mad3, or single-copy protein rescues of these deletions to wild-type strains and found that cells lacking Bub1 have a strong fitness defect when growing on benomyl while cells lacking Mad3 have a more moderate defect (*s*<-0.3 and *s* = -0.01317 respectively, Fig 4A). Remarkably, cells lacking both Bub1 and Mad3, but expressing the single-copy protein at the Bub1 locus, retain wild-type growth rate when challenged with the same benomyl concentration (*s* = 0.002, Fig 4A). Thus, even at the much higher resolution of these quantitative competition experiments, we find no evidence that the duplication and reorganization of the Bub1 and Mad3 proteins (Fig 1) confers a fitness benefit.

### No evidence of fitness advantage for the subfunctionalization of the Bub1/Mad3 ancestral protein

The experiments above show that there is apparently no fitness advantage to the Bub1/Mad3 protein organization relative to the single-copy protein. However, the real evolutionary trajectory almost certainly did not replace the single-copy protein directly with the fully formed Bub1 and Mad3. Instead, a series of evolutionary events (likely separated by millions of years) probably occurred from the single-copy protein to the extant paralogs. It is possible that certain steps along this path, particularly at the beginning after the duplication, were advantageous and drove the reorganization of the paralogs.

**Fig 5:**
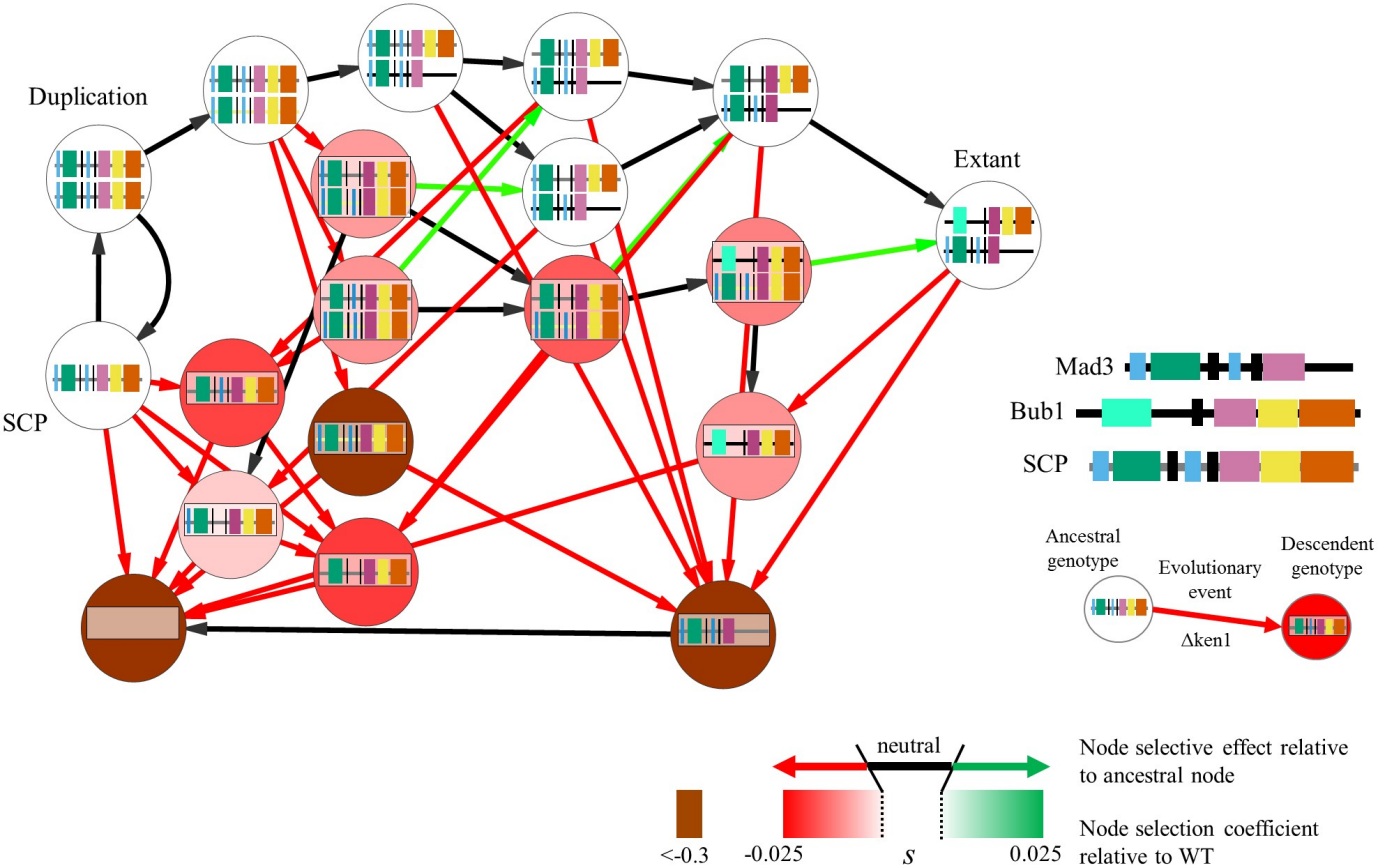
Evolutionary paths during network rewiring. Schematic of the intermediates we have assayed in our experiment if we assume changes in the MAD3 promoter occurred first following the gene duplication event. When the compared genotypes (nodes) are separated by a single mutational event (such as a degeneration of a functional element, which are shown as colored boxes inside the nodes), these nodes are connected by directed edges in the graph (see the example given for a loss of the first KEN box). The color of the node represents the fitness of the genotype when compared to wild-type. The color of the edge corresponds to the fitness of the descendent genotype when compared to the ancestral genotype, where red indicates a deleterious selection coefficient, black a selection coefficient with undetectable fitness effect, and green a positive selection coefficient. The color of the line representing the protein is grey for the SCP under the Bub1 promoter, yellow for the SCP under the Mad3 promoter, and black for the extant *S*. *cerevisiae* Mad3 and Bub1. Coloured boxes represent functional elements within the proteins (See Fig 1 and text for details).

Because there is insufficient phylogenetic resolution to infer the exact order and number of mutations, based on previous knowledge of the functional elements found in Bub1 and Mad3 (Fig 1), we created strains with genetic make-up of possible evolutionary intermediates that correspond to stepwise mutational events during protein function reorganization. The evolutionary paths assayed include mutations at multiple loci, and therefore possible paths were created using several rounds of the SGA cloning strategy (see Methods). Briefly, we sought to test single and double mutations of the KEN boxes on the SCP at the *BUB1* locus, loss of kinase of the SCP at the *MAD3* locus, non-functionalization of the gene, or “evolution” to the extant protein. These mutations represent key evolutionary steps from the ancestral protein to the extant Bub1 and Mad3 (Fig 5). We introduced these mutations into the SCP and cloned them individually into different starting strains (for example, we generated a strain containing the SCP with a mutated KEN box at the *BUB1* locus). To combine them, query strains carrying the SGA markers and different fluorophores were crossed to the library in an ordered array and selected such that the final products of several rounds of mating were otherwise genetically identical haploid spores carrying different combinations of marked alleles. We then performed an all-by-all competitive fitness assay, and found that genotypes cluster in three distinct fitness classes, which correspond to cells lacking Mad3 function (Δmad3-like, with an average selection coefficient of -0.015 relative to wild-type), cells lacking Bub1 function (Δbub1-like, with an average selection of <-0.3 relative to wild-type (fitness effects larger than -0.3 are not measurable in this assay and so we consider these genotypes to have fitnesses <-0.3, see Methods), or cells with wild-type phenotype (WT-like, with an average selection coefficient of 0.0026 relative to wild-type) (Fig 4B). Although we have not explored the fitness landscape exhaustively, this sample of genotypes suggests that it is made up of three distinct plateaus.

To assay possible paths through the fitness landscape that lead to sub-functionalization, we focused on genotypes that differed from each other by one ‘evolutionary step’. For example, we consider the initial duplication as one evolutionary step (such that we compared the fitness difference of a strain containing one vs two SCP), as well as individual losses of functional motifs. Although in principle it is possible for a non-functional genotype to revert, we consider these events to be rare and therefore have not considered them for simplicity. The results of our analysis are displayed in Fig 5, as an evolutionary landscape with multiple evolutionary paths that ‘travel’ through possible intermediate genotypes. Interestingly, we were able to find at least one path without a detectable fitness defect consisting of at least three degenerations (Fig 5, path through white nodes). If we only consider paths that do not go through nodes of fitness defects (i.e. we do not allow crossing of fitness valleys), then the analysis suggests that the extant network in *S*. *cerevisiae* is an absorbing state (see Discussion).

**Fig 6:**
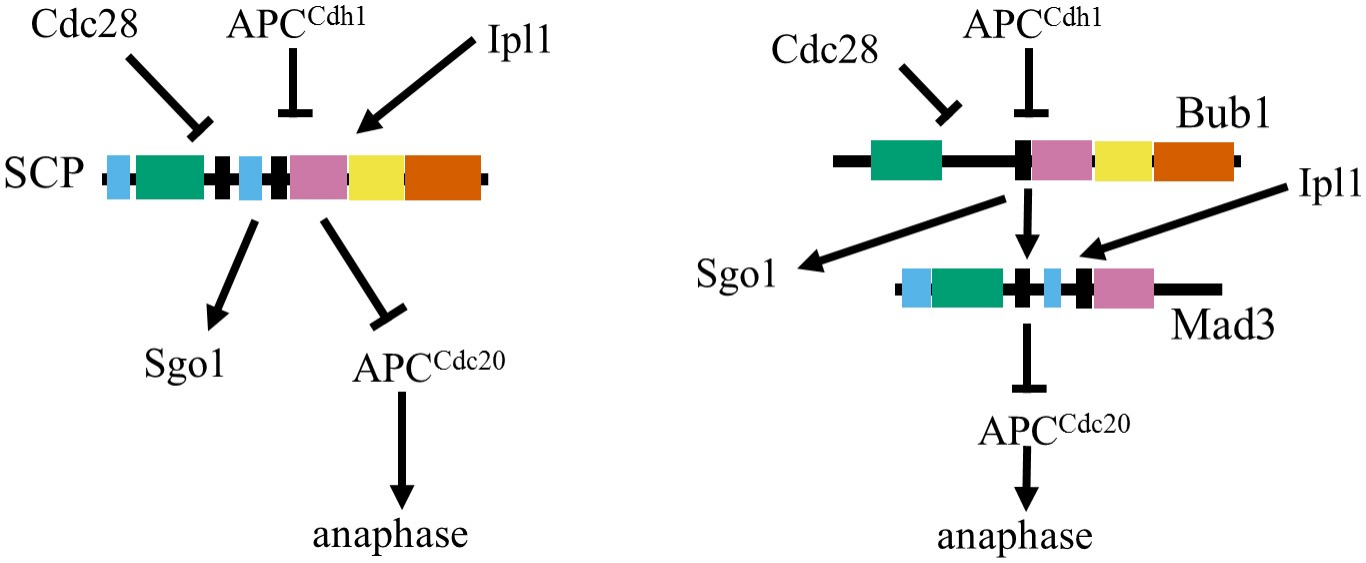
Model for network rewiring. On the left is a schematic of the inferred ancestral spindle checkpoint with the single-copy protein (SCP is the genotype marked as *L*. *kluyveri* in Fig 1), while on the right is a schematic of the extant *S*. *cerevisiae* network (genotype same as marked in Fig 1).

Although we cannot be certain that evolution has taken any of the paths studied here, that we can find at least one seemingly neutral evolutionary path, at the resolution of our assay, strongly supports the DDC hypothesis that the reorganization of protein domains in the Bub1/Mad3 paralogs can be explained by neutral degenerative mutations (Fig 6 and see Discussion).

This model also predicts that the initial neutral step in the process (the duplication) is reversible, and that the mutations must be relatively common to explain the frequency at which the reorganization occurs during evolution (Fig 1). Consistent with this, we have identified at least one phylogenetic clade where the gene duplication in the ancestor of Saccharomyces reverted to the single-copy functional homolog: *Vanderwaltozyma polysporus* retains a single-copy protein with all the functional elements of Bub1 and Mad3 even though it diverged after the whole-genome duplication (S4 Fig). It is estimated that the ancestor to the lineages leading to *V*. *polysporus* and *S*. *cerevisiae* had already non-functionalized ~20% of the duplicates [21] suggesting that neither sub-functionalization of Bub1/Mad3 and non-functionalization were rapid.

## Discussion

Neutral processes, such as described by the DDC model [7], have been shown to be important in increasing genomic complexity (see for example [22] for a study on non-adaptive increase in interactome complexity). At the limit of the resolution of our assay (discussed below), we find no evidence that the Bub1/Mad3 protein reorganization after duplication in budding yeast provides a route for adaptive conflict resolution and therefore, further work is necessary to find a fitness advantage over a single-copy protein in the spindle checkpoint pathway.

Consistent with the DDC model, none of the evolutionary intermediates that we consider functional through the comparative genomics analysis showed a fitness defect when tested under laboratory conditions requiring functional spindle checkpoint and when driven by the *BUB1* promoter. Nevertheless, there are several caveats to our experiment. First, the resolution of our assay meant that we could only detect fitness effects in the range of *s* = 0.005. Because of the population size of budding yeasts in nature [23] and our estimated effective population size during the experiment (see Methods), selection could be efficient even on undetected differences in fitness (given a high enough recombination rate). Therefore, it remains possible that the sub-functionalization to Bub1/Mad3 is truly adaptive. However, even if we assume a very small beneficial effect that was not detected, because we performed our assay in the presence of high concentration of benomyl (which was used to characterize all the components of the spindle checkpoint pathway [24]), we believe that under reasonable growth conditions the adaptive effect of this sub-functionalization would be even smaller. Another possibility is that Bub1 and Mad3 participate in completely orthogonal molecular processes to the spindle checkpoint such that our assayed environment would not be able to detect the real functional differences between the duplicate genes and the ancestral single-copy protein. Yet a third possibility is that there exists one context where the effects of the duplication are under much stronger selection. However, if this context exists, it must be rare due to the finding that the phylogenetic clade leading to *V*. *polysporus* reverted to the single-copy gene. Ultimately, it is not possible to know the environmental context, nor the genetic context of the ancestral yeast and we cannot rule out that adaptation by escape of an adaptive conflict drove the sub-functionalization of the ancestral protein. Despite these caveats, we note that the functionally relevant motifs in Bub1 and Mad3 were identified in the context of the spindle checkpoint pathway, and this pathway is activated every cell division to ensure proper chromosomal attachment prior to anaphase. We therefore believe that large effects would have been captured even in this laboratory environment.

Although in our study all functional evolutionary paths lead to the same genotype through the same number of degenerations, the probability that a path is taken is dependent on the rate of mutation, the selection coefficient of the intermediates and the effective population size [25]. We here discuss only the scenario of large population size because small population sizes would allow all genotypes, including any potential neo-functionalization, to be effectively neutral. When population size is large and mutation rate is low, then crossing a fitness valley (as measured by our quantitative fitness assay) requires the population to fix each intermediate genotype (this is the deleterious sequential fixation regime discussed in [25]). In this large population size and low mutation rate regime, paths through deeper fitness valleys are essentially never taken because selection is very effective. In this regime, the relative frequencies of the effectively neutral states are entirely dependent on the mutational rate between these states [7]. Because the mutational rate to degenerate is likely to be higher than the mutational rate to re-create a functional element, the evolutionary outcome of Bub1/Mad3 homologs after sub-functionalization could be nearly deterministic. Therefore, the relative probability of observing Bub1/Mad3 functional homologs after duplication is equal to the rate of sub-functionalization divided by the rate of non-functionalization [7]. We propose that the rate of sub-functionalization can be high due to the very small number and mechanistically simple degenerations: we showed here that sub-functionalization and degeneration of the Bub1/Mad3 protein can occur within only two mutations following the gene duplication (generation of stop codon to remove the kinase function in Mad3, and a shift in start position to remove the first KEN box in Bub1), both of which have been observed frequently over evolution in other genes [26,27]. If this sub-functionalization is truly neutral, we propose that the repeated reorganization of the Bub1/Mad3 homologs may be due to the fact that no other possible outcome of the duplication can be easily observed (it is an absorbing state), such that the observed genotype is surrounded by fitness valleys or reversion to the single-copy protein. Fixation of the re-organization may have occurred through hitchhiking with other beneficial mutations, a scenario that often occurs with large population size and a high mutation rate [28], or due to changes in gene expression (discussed below).

Previous studies have considered the functional implications of evolution of individual enzymes and protein complexes through gene duplication and divergence (e.g. [29,30]). However, many proteins that function within regulatory networks contain complex multi-domain architectures and disordered regions [31]. In the case of Bub1 and Mad3, numerous functional steps occurred after gene duplication, and it is not possible to reconstruct them at the resolution of single amino acid substitution. Nevertheless, we chose several key evolutionary steps during the functional reorganization and assessed the fitness of those intermediates. We believe that our study represents a practical way forward for studies of the evolution of complex eukaryotic proteins. Unlike other previous studies (e.g. [29]), we have not performed ancestral gene resurrection via gene synthesis, but instead we have chosen a gene from another species which we believe is representative of the ancestral allele. Our approach has several advantages. First, it is more likely that the gene is functional in at least one genetic environment. Second, the reconstruction of the ancestral gene might not be accurate in proteins with highly diverged disordered regions. Finally, the gene has evolved for the same period of time as the duplicate genes, providing a direct test of whether adaptation from the ancestral allele is due to the resolution of an adaptive conflict. Similar experimental designs have been used to assay functional differences in whole transcriptional regulatory networks that happen on relatively shorter divergence times [32].

Transcriptional evolution has been shown to be an important aspect of functional divergence after gene duplication [33–35]. An important caveat of our study is that we have not tested the evolution at the promoter, due to the difficulty in finding the functional elements of the promoter region [36]. However, we do have evidence that the Mad3 promoter and the Bub1 promoter are not functionally equivalent as the single-copy protein does not fully rescue the spindle-checkpoint defects when placed at the *MAD3* locus (S5 Fig). Those promoter changes, however, are still consistent with a model of degeneration where the Bub1 promoter is similar to the ancestral SCP promoter. On the other hand, it is also possible that after duplication the Bub1 promoter acquires beneficial mutations relative to the ancestral promoter, and these changes drive fixation of the gene duplication. Thus, although there is clear evidence that the promoters of Mad3 and Bub1 have diverged, we did not test the effects of these changes.

Under our tested conditions, our study shows that there is no evidence that the reorganization of protein function was to escape an ‘adaptive conflict’ for the spindle checkpoint pathway in mitotic cells of *S*. *cerevisiae*. Our data is consistent with the DDC model and our study suggests that parallel evolution through degenerative processes does not have to be rare or adaptive. Further work will be required to see if this is also the case in other organisms where this reorganization has been observed. This situation is reminiscent to the convergent evolution of holocentric chromosomes across the tree of life [37], and it is still unclear whether it provides an advantage during growth, especially considering the more complex meiotic segregation of chromosomes [38].

Interestingly, although the core spindle checkpoint pathway is conserved in all eukaryotic life, several other differences exist in this pathway [39]. These differences include non-conserved proteins important for spindle checkpoint function (such as p31) or different copy number of paralogous proteins (such as Cdc20 in human). Our study provides experimental techniques to test the step-wise effects of evolutionary changes that have been detected through comparative genomics on multiple loci (such as the ones in the spindle checkpoint). We anticipate that these sensitive quantitative fitness measurements will be useful in the computational modeling of sequence and protein evolution within the context of a complete regulatory network [40,41], as has been performed on other important regulatory networks [42,43].

## Materials and methods

### Yeast strains and culturing

All strains were derived from either BY4741 or BY4742 using standard yeast genetic techniques or synthetic gene arrays (see next method subsection). The single-copy gene was PCR amplified from purified genomic DNA (Fermentas, #K0512) of *L*. *kluyveri* (NRRL Y-12651). All integrations were verified by PCR, and key strains containing the single-copy gene were verified for absence of Bub1 or Mad3 when relevant.

Strain construction for the library of alleles was performed using the same method as described in [44]. SGA query strains were created by transferring the Ste3pr_LEU2 marker from Y8205 into the *CAN1* locus of BY4742. Fluorophores with the Ste2pr_LkHIS3 were cloned into the pAN200a plasmid (based on pFA6a [45]) using standard cloning techniques and transformed into the *CAN1pr* locus using delitto perfetto [46].

Benomyl (10mg/mL DMSO stock) is used at outlined concentrations and added to boiling-hot media until completely dissolved. 5-fluoroorotic acid (5-FOA, 100mg/mL DMSO stock) was used to select against uracil biosynthesis prototrophs [47] and plates were poured at 1g/L 5-FOA final concentration (supplemented with all amino acids, including 72ug/mL uracil). Geneticin (G418) was used at 200ug/mL to select for geneticin resistance.

### Synthetic genetic arrays

Cells are grown according to slight modifications to the protocols outlined in [48] as shown as a schematic in S3 Fig. We modified our query strains to have the following cassette integrated at the *CAN1* locus: RPL39pr_fluorophore_Ste2pr_LkHIS3_Ste3pr_LEU2. Fluorophores used for our study were yeast-enhanced monomeric green fluorescent protein (ymEGFP) and yeast-mCherry (ymCherry). For the general construction of our strains, a query strain is first crossed with all the desired alleles at a particular locus. Diploid selection is performed by selecting for complementary auxotrophies. Overnight diploid cells from plate patches are then scraped into liquid sporulation media (1% Potassium acetate, 0.005% Zinc acetate) and supplemented with amino acid requirements for diploid strains at 25% of the normal usage and incubated on a roller wheel for three days at room temperature. Usually, about 30% sporulation is observed and 5ul of the mixture is spread or spotted on selection plates that select for MATα and other selection markers such as auxotrophies and drug markers.

We found that modifications to the germination and outgrowth procedure were necessary in our hands to obtain colonies after the SGA procedure. In our hands, addition of lysine to the media greatly enhances the initial outgrowth of spores for all strains that were constructed using our query strains (even the ones that did not express a fluorophore or were lysine prototrophs). Spore outgrowth was normal for standard SGA query strains, indicating that strain specific variation, or heterozygous *lys2* deletion strains or homozygous *LYP1* affected the outgrowth of our strains (*LYS2*/*Δlys2 LYP1*/*LYP1* compared with *LYS2*/*LYS2 LYP1*/*Δlyp1*). Therefore, to select for lysine prototrophs, the colonies are replicated to media lacking lysine only after the initial growth. To select for lysine auxotrophy, replica plating was used to isolate colonies that did not grow on media lacking lysine, however alpha-aminoadipate could be used instead [49].

The final strains used in the competitive fitness assay all had the following genotypes: MATα, can1::RPL39pr_fluorophore_Ste2pr_LkHIS3_Ste3pr_LEU2, bub1::allele::CaURA3MX, mad3::allele::KanMX, Δlys2, Δhis3, Δura3, Δleu2.

### Genomic sequences and comparative analyses

Protein sequences used for the comparative analyses were from the Yeast Gene Order Browser [50]. These proteins were then aligned with MAFFT [51]. Genomic sequences for BY4741 and BY4742 were obtained from the Saccharomyces Genome Database [52].

To perform our comparative analyses, protein sequences were analyzed using methods described in [9] and by visual inspection. To test for changes in constraints in the duplicate protein, we first predict regions of conservation in the proteins pre-duplication using a phylogenetic hidden Markov model that detects regions that have significantly lower evolutionary rates relative to their flanking region [53]. Having predicted these conserved regions, we can then map them to the duplicate protein and ask whether two rates of evolution (one for the pre-duplication clade, and one post-duplication) better explain the evolution of the selected region as opposed to a single rate. Because the rate of evolution of predicted conserved regions in the ancestral protein is very low, when two rates of evolution better explain the data, it typically implies that the region under purifying selection prior to the whole-genome duplication is now under relaxed constraints after the whole-genome duplication. Partitioning of these losses in selection constraints in the two paralogs is an indication of sub-functionalization. To identify potential new motifs in the post-duplication proteins, the phylogenetic hidden Markov model can be used on the post-duplicate protein and the same analysis for changes in constraints can be performed.

X-ray crystallography files (3ESL: Bub1 [54] and 4AEZ: Mad3 [11]) were obtained from the Protein Data Bank [55] and analyzed using PyMOL [56].

### Chromosome loss assay

Chromosome loss rate was measured in the classical strain carrying a linear non-essential chromosome in the W303 background [16]. Briefly, strains carrying the ochre allele *ade2-101* mutation exhibit a visible red pigment when growing in media with low adenine supplementation. This phenotype is suppressed in strains with a single-copy functional *SUP11*, which is present in the artificial chromosome. Therefore, chromosome loss rate can be measured by counting the number of red-sectored colonies in agar plates containing low adenine. This assay is performed by plating about 200 colonies, and we report the number of sectored colonies over the number of white colonies after two days of growth.

### Quantitative fitness assay

Quantitative fitness assays were performed using the MACSQuant VYB (Miltenyi Biotec Inc.). Briefly, strains are grown for 48 hours in 5mL of cultures on a rolling wheel. The competitive fitness experiment is started by mixing relatively equal proportion of green cells and red cells in deep 96-well blocks (100ul of a single ymCherry expressing strain and 100ul of a single ymEGFP expressing strain into 600ul distilled water) at a 4-fold dilution. To obtain further dilutions, 20ul of the competition is diluted into 300ul distilled water to form a 16 fold dilution, then 20ul of this dilution is diluted into 300ul of defined media supplemented with amino acids and 100ug/mL ampicillin to form a final 16-fold dilution. The cells are therefore diluted 1024-fold and this operation is performed every 24 hours. Given a conservative estimate of 2*10^8^ yeast cells per mL at saturation, we estimate an effective population size (Ne) of approximately 3.44 * 10^5^. Each screen competes 8 genotypes against the others (64 wells), with an additional 16 wells used as contamination control. We use the diagonal of the competitions as an additional negative control as these were competing genetically identical strains constructed independently with different fluorophores. Competitive fitness assays were performed in synthetic media with 10ug/mL benomyl, which is a condition at which wild-type cells still undergo ten divisions per day but seriously impairs growth of cells lacking spindle checkpoint function.

We defined the relative selection coefficient (*s*) on the basis of the deterministic continuous time model of logistic growth of an allele against another: dR/dt = sRG, where R and G are the frequencies of red and green cells in the population, t the number of generations, and s the selection coefficient [57]. To simplify the parameter inference, we ignore drift (because the timescale of the experiment is very short compared to Ne), mutational processes that happen during the fitness assay (i.e. we ignore mutations occurring in red or green cells that may alter the lineage trajectories), recombination, and any non-transitive effects. Constraining on G = 1-R, this model describes the logistic equation and has solution [57]:

$$R\left(t\right)=\frac{R\left(0\right)e^{st}}{\left(1-R\left(0\right)\right)+R\left(0\right)e^{st}}$$

Because we do not observe the frequencies directly, we estimate the parameters s (and R(0) if needed) by counting each cell sampled by the flow-cytometer as a random variable, taking values of red or green (as it is described for a binomial logistic regression). In total, 50 000 cells are analyzed at the 20^th^ and the 40^th^ generation for each competition experiment and well-defined proportions of green and red cells are gated to remove doublets and relative cell counts are obtained from each competition [58,59]. The likelihood function for this counting process is simply:

$$\text{L}=\overset{T}{\underset{t=1}{\prod }}\frac{n_{t}!}{r_{t}!\left(n_{t}-r_{t}\right)!}R{\left(t\right)}^{r_{t}}{\left(1-R\left(t\right)\right)}^{n_{t}-r_{t}}$$

Where t are the time points, r the number of red cells counted at that time point, n the total number of cells counted at that time point and R(t) the expected frequency of red cells at time point t. We estimate the initial frequency R(0) and s (due to their relationship with R(t)) by maximizing the likelihood, and the maximum likelihood estimate for the selection coefficient can be obtained by performing a log-linear regression on the ratio of red and green cells in the population:

$$\text{log}\left(\frac{R\left(t\right)}{G\left(t\right)}\right)=\text{log}\left(\frac{R\left(0\right)}{G\left(0\right)}\right)+st$$

For more than two time points, the parameters can be estimated by an iterative reweighted least-squares linear regression or with Newton’s method. For two time points, the solution is equivalent to the simple linear regression and we use the same formula as in [59]:

$$\frac{\text{log}\left(\frac{r\left(t2\right)}{g\left(t2\right)}\right)-\text{log}\left(\frac{r\left(t1\right)}{g\left(t1\right)}\right)}{t2-t1}=s$$

Clearly, if *s* is positive, then the ratio of red cells to green cells increases at the next generation and the value of the selection coefficient is therefore the selective effect of a beneficial allele as in [60].

The upper limit of detection occurs when we expect fewer than 1 out of 50000 cells of the worst genotype at the 40^th^ generation, which occurs at approximately *s* = -0.3. In practice, some genotypes can no longer be detected at the 20^th^ generation, and we simply report these as *s* < -0.3. In some other cases, the number of red or green cells is fewer than 50, which leads to highly inaccurate estimates of the selection coefficient and we also report these as *s* < -0.3. When the total number of cells counted for both strains in a competition was fewer than 50, we reported *s* = 0 to mean equally lethal.

All genotypes were made in strains expressing red or green fluorescent proteins, and therefore assayed in two biological replicates. Key genotypes with undetectable fitness effects were further assayed with at least two technical replicates (where the same strains were assayed more than once on different days).

### Localization analysis

Cells were grown in low-fluorescence media with appropriate auxotrophic requirements to log-phase and imaged using a Leica SP8 confocal microscope. Proteins of interest were tagged with EGFP [61], while the cytoplasm of cells were marked with cytoplasmic mCherry or mTagBFP2 [62] under a constitutive ribosomal promoter. We imaged the green fluorescence first, followed by the cytoplasmic marker to prevent bleaching. This setup enables highly controlled and quantitative analysis of localization because strains with differently tagged proteins can be imaged on the same field of view under identical conditions.

Double-blind quantification of the localization pattern was performed by manually inspecting the green fluorescence localization pattern first and scoring for whole nucleus, kinetochore (punctae), or a mixture of both. The scored cells were then assigned their proper genotype by looking at the red or blue fluorescence channels.

## Supporting information

S1 Fig Amino acid resolving power of changes in constraints are biologically relevant. A) Alignment of an example change in constraint in the Bub1 lineage. Numbers indicate residue position within the *S*. *cerevisiae* Bub1 protein. Alignment within the blue rectangle represents in the first N-terminal KEN box, while in green represents a subset of the TPR domain. B) Structural alignment of the region shown in A). The TPR domain of Bub1 (green) and Mad3 (cyan) are shown, along with the N-terminal tail of Mad3. Regions highlighted in red, orange and yellow correspond to regions boxed in A). C) Schematic representation of the binding interaction of Mad3 with Cdc20 can help elucidate why the regions shown in A) have correlated evolution. D) Alignment of a change in constraint in the TPR of Bub1 in independent duplication events.
(TIF)

S2 Fig Effect of benomyl on the growth of other yeast species. Growth of *S*. *cerevisiae* cells of different genotypes is impaired at high benomyl concentration, while other yeast species are relatively unaffected as assessed by a 10-fold spot dilution assay. YPD plate was imaged after 2 days. YPD plate containing 30ug/mL benomyl was imaged after 3 days.
(TIF)

S3 Fig Schematic of the Synthetic Genetic Array (SGA) methodology. Query strains containing SGA mating type reporters and fluorescent proteins are sequentially crossed to an ordered array of yeast strains containing marked alleles at different loci. The initial cross introduces alleles belonging to one locus, and the product of the haploid selection process after sporulation produces new query strains that differ by the allele introduced at one locus. The alleles at the second locus are then introduced in a second cross. Pink yeast outlines represent the MATα mating type, while blue yeast outlines represent the MATa mating type.
(TIF)

S4 Fig Reversion to single-copy protein in the *Vanderwaltozyma* clade. A) Schematic of the critical functional regions of the proteins homologous to the SCP in the *Vanderwaltozyma* clade, which is also a post-whole-genome duplication species, suggesting a reversion to SCP after gene duplication. B) Alignment of the critical functional regions of the single *Vanderwaltozyma polysporus* homologous protein to the SCP (L. klu), to Bub1 and Mad3 from *S*. *cerevisiae*.
(TIF)

S5 Fig Effect of the MAD3 promoter in the expression of the single-copy protein. Spot dilution assay showing that the SCP does not rescue the spindle checkpoint defect of cells lacking Mad3 when placed at the MAD3 locus (under the MAD3 promoter). YPD plate containing 25ug/mL benomyl was imaged after 3 days.
(TIF)
