# Supplementary figures and images for: Parallel reorganization of protein function in the spindle checkpoint pathway through evolutionary paths in the fitness landscape that appear neutral in laboratory experiments
Source: PLoS Genet. 2017 Apr 14;13(4):e1006735. doi: 10.1371/journal.pgen.1006735 (PMC5409178; doi:10.1371/journal.pgen.1006735)

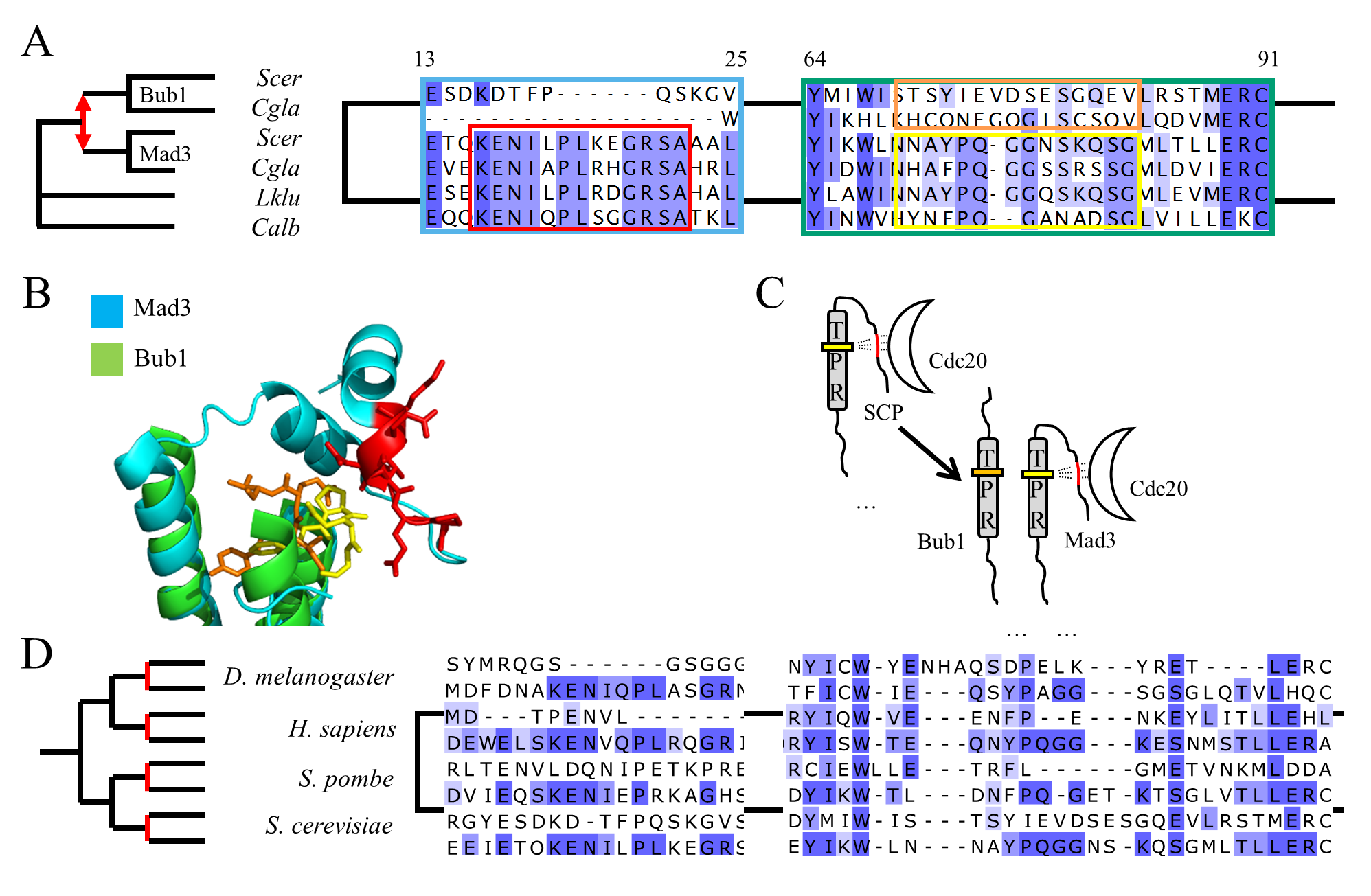

Supplement: S1 Fig — A) Alignment of an example change in constraint in the Bub1 lineage. Numbers indicate residue position within the S. cerevisiae Bub1 protein. Alignment within the blue rectangle represents in the first N-terminal KEN box, while in green represents a subset of the TPR domain. B) Structural alignment of the region shown in A). The TPR domain of Bub1 (green) and Mad3 (cyan) are shown, along with the N-terminal tail of Mad3. Regions highlighted in red, orange and yellow correspond to regions boxed in A). C) Schematic representation of the binding interaction of Mad3 with Cdc20 can help elucidate why the regions shown in A) have correlated evolution. D) Alignment of a change in constraint in the TPR of Bub1 in independent duplication events. (TIF) [file pgen.1006735.s001.TIF]

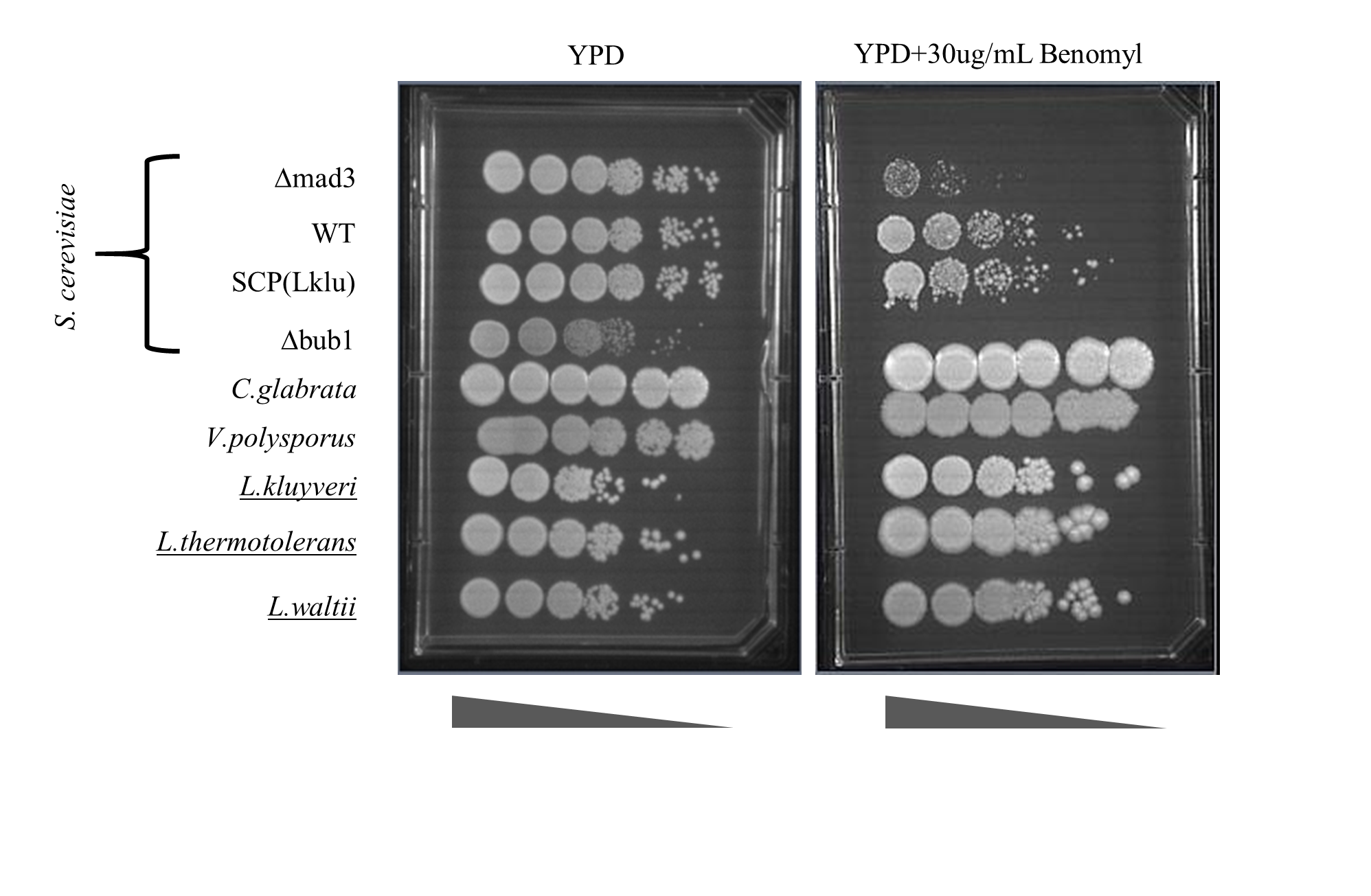

Supplement: S2 Fig — Growth of S. cerevisiae cells of different genotypes is impaired at high benomyl concentration, while other yeast species are relatively unaffected as assessed by a 10-fold spot dilution assay. YPD plate was imaged after 2 days. YPD plate containing 30ug/mL benomyl was imaged after 3 days. (TIF) [file pgen.1006735.s002.TIF]

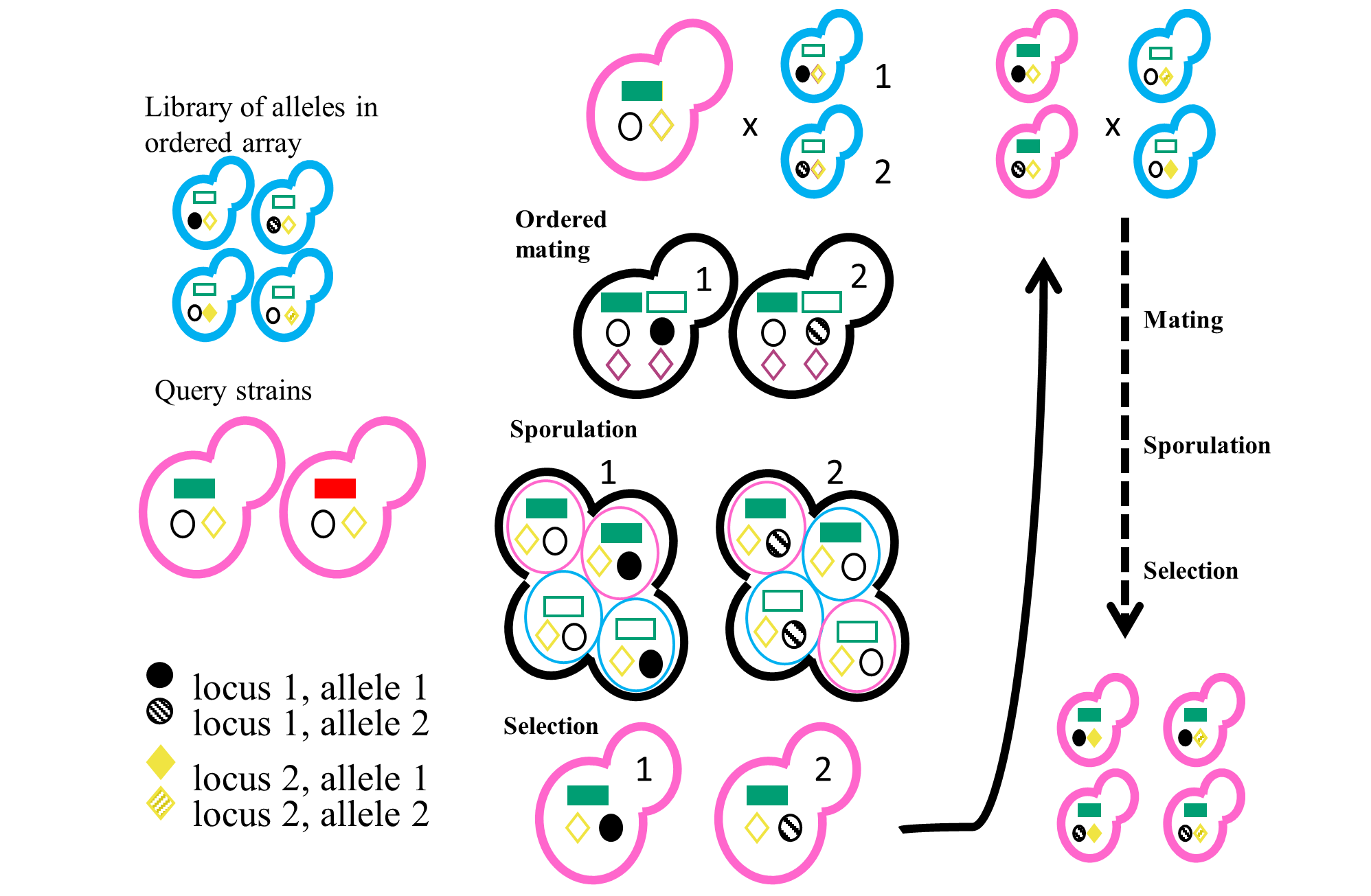

Supplement: S3 Fig — Query strains containing SGA mating type reporters and fluorescent proteins are sequentially crossed to an ordered array of yeast strains containing marked alleles at different loci. The initial cross introduces alleles belonging to one locus, and the product of the haploid selection process after sporulation produces new query strains that differ by the allele introduced at one locus. The alleles at the second locus are then introduced in a second cross. Pink yeast outlines represent the MATα mating type, while blue yeast outlines represent the MATa mating type. (TIF) [file pgen.1006735.s003.TIF]

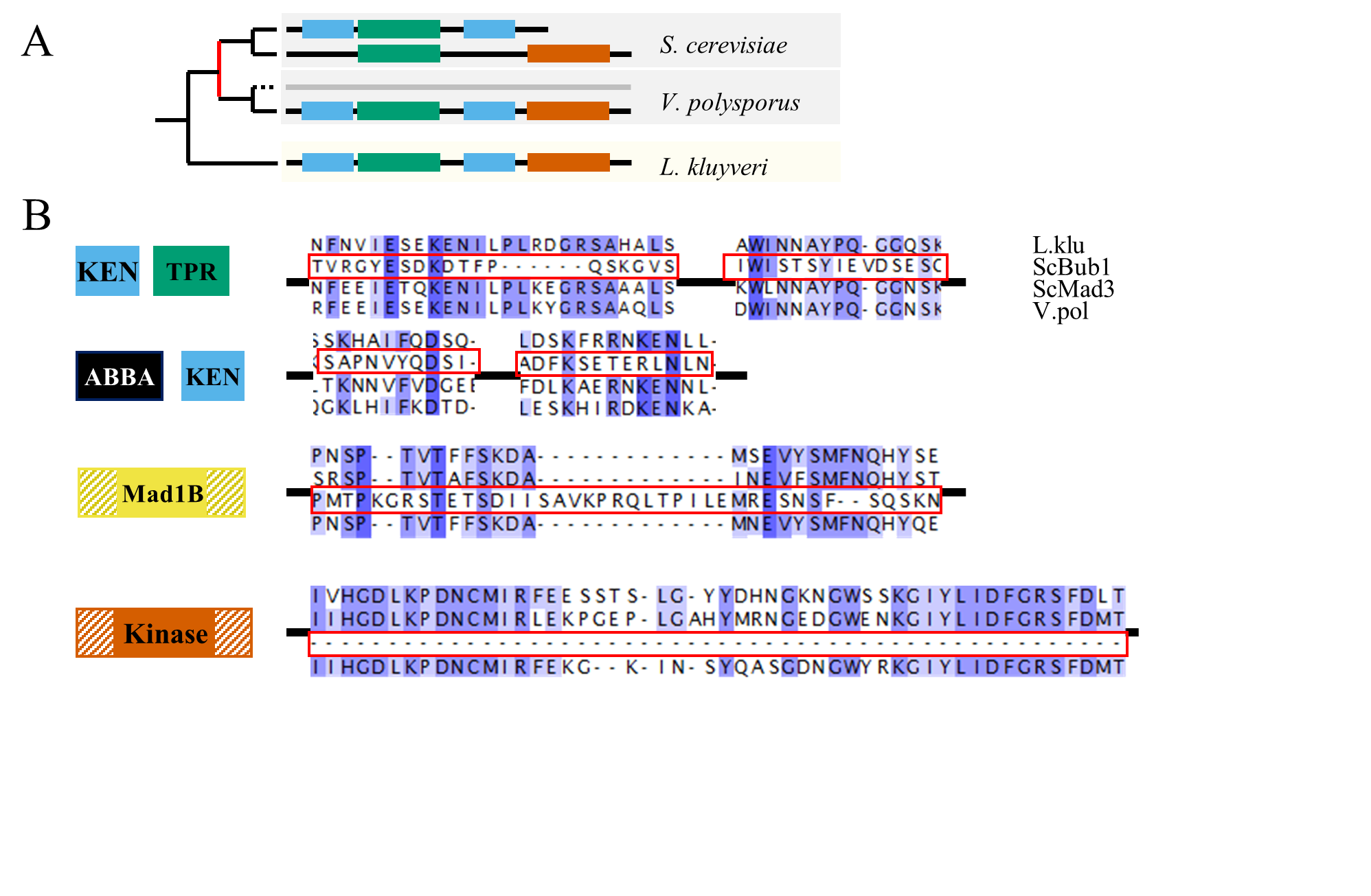

Supplement: S4 Fig — A) Schematic of the critical functional regions of the proteins homologous to the SCP in the Vanderwaltozyma clade, which is also a post-whole-genome duplication species, suggesting a reversion to SCP after gene duplication. B) Alignment of the critical functional regions of the single Vanderwaltozyma polysporus homologous protein to the SCP (L. klu), to Bub1 and Mad3 from S. cerevisiae. (TIF) [file pgen.1006735.s004.TIF]

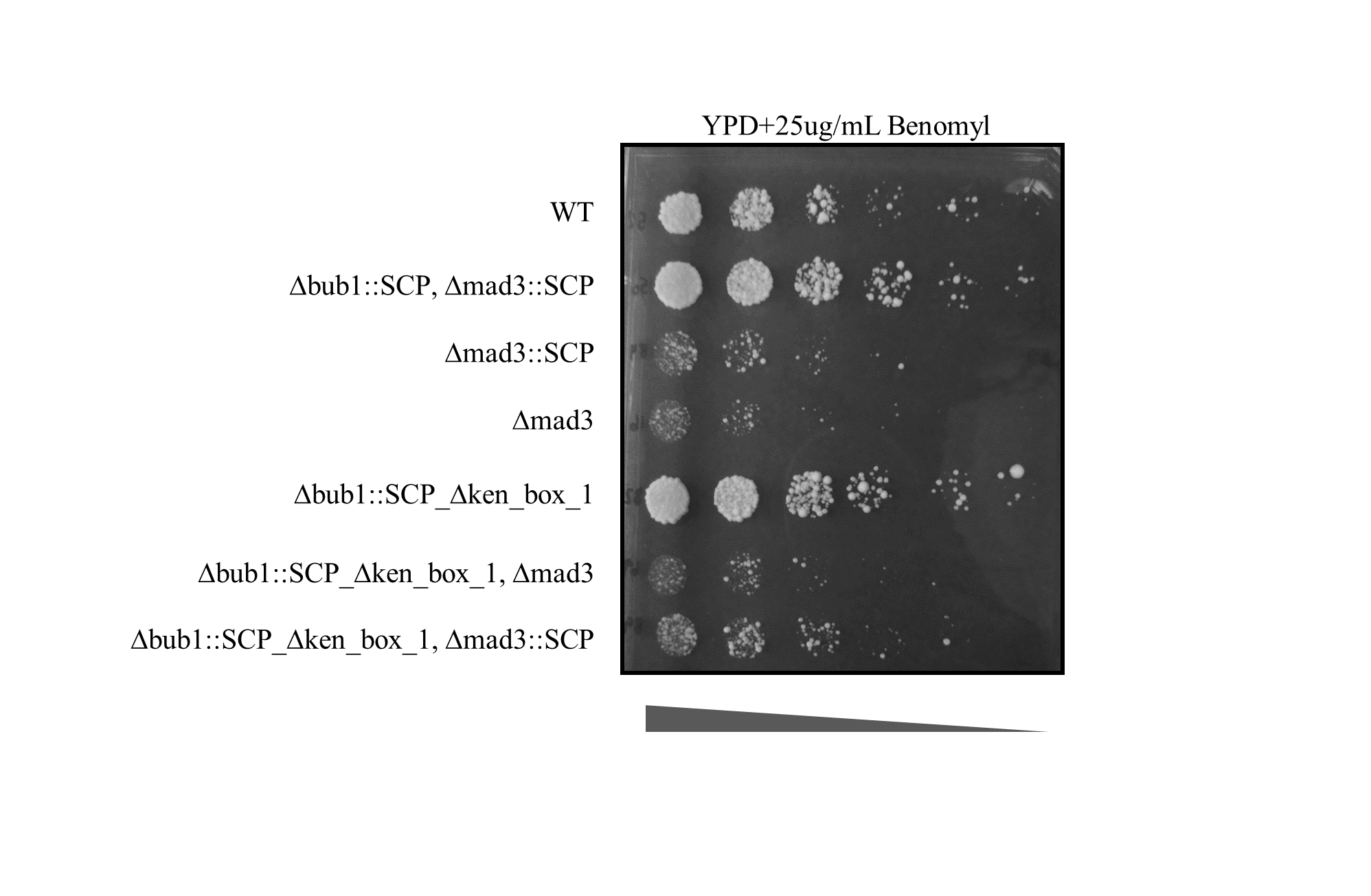

Supplement: S5 Fig — Spot dilution assay showing that the SCP does not rescue the spindle checkpoint defect of cells lacking Mad3 when placed at the MAD3 locus (under the MAD3 promoter). YPD plate containing 25ug/mL benomyl was imaged after 3 days. (TIF) [file pgen.1006735.s005.TIF]
